# Effects of esomeprazole treatment for gastroesophageal reflux disease on quality of life in 12- to 17-year-old adolescents: an international health outcomes study

**DOI:** 10.1186/1471-230X-9-84

**Published:** 2009-11-18

**Authors:** Thirumazhisai Gunasekaran, Vasundhara Tolia, Richard B Colletti, Benjamin D Gold, Barry Traxler, Marta Illueca, Joseph A Crawley

**Affiliations:** 1 Lutheran General Children's Hospital, 1675 Dempster Street, Park Ridge, IL 60068, USA; 2 Loyola Medical Center, 2160 South 1st Street, Maywood, IL 60153, USA; 3 Wayne State University, 4743 Cass Avenue, Detroit, MI 48201, USA; 4 University of Vermont, 201 South Prospect Street, Burlington, VT 05401, USA; 5 Emory University School of Medicine, 1648 Pierce Drive, Atlanta, GA 30322, USA; 6 AstraZeneca LP, 1800 Concord Pike, Wilmington, DE 19803, USA; 7 Providence Hospital, Southfield, MI, USA

## Abstract

**Background:**

Although gastroesophageal reflux disease (GERD) is common in adolescents, the burden of GERD on health-related quality of life (HRQOL) in adolescents has not been previously evaluated. Therefore, the objective of the study was to examine the effect of GERD on HRQOL in adolescents.

**Methods:**

This international, 31-site, 8-week safety study randomized adolescents, aged 12 to 17 years inclusive, with GERD to receive esomeprazole 20 or 40 mg once daily. The Quality of Life in Reflux and Dyspepsia questionnaire (QOLRAD), previously validated in adults, consists of 25 questions grouped into 5 domains: emotional distress, sleep disturbance, food/drink problems, physical/social functioning, and vitality. The QOLRAD was administered at the baseline and week-8 (final) visits.

**Results:**

Of the 149 patients randomized, 134 completed the QOLRAD at baseline and final visits and were eligible for analysis of their HRQOL data. Baseline QOLRAD scores indicated GERD had a negative effect on the HRQOL of these adolescents, especially in the domains of vitality and emotional distress, and problems with food/drink. At the final visit, mean scores for all 5 QOLRAD domains improved significantly (*P *< .0001); change of scores (ie, delta) for all domains met or exceeded the adult QOLRAD minimal clinically significant difference standard of 0.5 units.

**Conclusion:**

GERD had a negative effect on QOL in adolescents. After esomeprazole treatment, statistically and clinically significant improvements occurred in all domains of the QOLRAD for these adolescents.

**Trial Registration:**

D9614C00098; ClinicalTrials.gov Identifier NCT00241501

## Background

Gastroesophageal reflux disease (GERD) is recognized increasingly as a common condition in adolescents [1]. Recent surveys of high school students show that at least 21% had significant GERD symptoms occurring a minimum of 1 time per month [2-4]. A survey conducted in pediatric practices revealed that 5.2%, 5.0%, and 8.2% of children and adolescents aged 10 to 17 years reported experiencing heartburn, epigastric pain, and regurgitation, respectively, in the previous week [5]. Moreover, in the same time period, 27.9% of children aged 10 to 17 years experienced abdominal pain, which may be a symptom of GERD [5].

Findings of numerous studies have shown the negative effect of GERD on health-related quality of life (HRQOL) in adults; however, few studies have assessed the effect of GERD on HRQOL in children and adolescents [6-15]. Although previously validated in adults [16], no data are available on the validity and performance of the Quality of Life in Reflux and Dyspepsia questionnaire (QOLRAD) in adolescent GERD patients or on the burden of GERD on HRQOL in adolescents.

Proton pump inhibitors (PPIs) have been recommended as the most effective acid suppression therapy for adults and children with GERD [17,18]. PPIs have been shown to relieve or resolve GERD symptoms in most adult and pediatric patients [19-23]. In Gold et al, we reported the safety and efficacy of esomeprazole in treating the symptoms of GERD in adolescents [23]. In the current article, we explore the effects of esomeprazole on QOL in the adolescent patients from the Gold et al [23] study.

## Methods

### Study design

This international, phase 3, randomized study double blinded for dose safety was conducted at 31 sites in Canada, France, Italy, and the United States (D9614C00098; ClinicalTrials.gov Identifier NCT00241501). The study was performed in accordance with the ethical principles that have their origin in the Declaration of Helsinki and that are consistent with ICH/Good Clinical Practice. The study protocol was approved by the institutional review boards, and all patients and their parents or guardians provided written informed consent and assent before any study procedure was conducted.

Patients were randomized 1:1 to receive esomeprazole (Nexium^®^; AstraZeneca LP, Wilmington, DE) 20 or 40 mg (no placebo control) orally once daily for 8 weeks. Esomeprazole was administered approximately 60 minutes before breakfast. For patients unable to swallow capsules, capsule contents could be mixed with 1 tablespoon of applesauce. Age-appropriate chewable antacid tablets were provided for use as rescue medication.

### Patients

Adolescents aged 12 to 17 years with a clinical diagnosis of GERD based on medical history, physical examination, any laboratory test results, and/or information from any diagnostic testing (eg, pH monitoring, endoscopy, biopsy) were eligible to participate in the study. The study entry criteria were consistent with GERD diagnostic guidelines of the North American Society for Pediatric Gastroenterology, Hepatology, and Nutrition [17]. Patients who were infected with *Helicobacter pylori *(as documented by the investigator using standard medical practice) but had no evidence of active ulceration or recent gastrointestinal bleeding were permitted at the discretion of the investigator. Postpubertal girls must have had a negative result on their urine pregnancy tests at the screening and week-4 and -8 visits. Patients who had any gastrointestinal pathology that required surgery, interfered with study participation, or potentially confounded study data were excluded. Patients who had an acute or chronic illness or condition (eg, pervasive developmental disorders, suspected abuse/neglect, recent trauma) that, in the opinion of the investigator, placed the patient at risk for not completing the study or for potentially confounding the study data were excluded. Patients could not have taken any PPI within 14 days or H_2_-receptor antagonist or prokinetic agent within 3 days of randomization. Patients also could not use any study-restricted medications (eg, antiemetics, bismuth-containing products, macrolide antibiotics, systemic steroids) on a continuous basis during the study.

### Assessments

The QOLRAD, previously validated in adults [16], generally can be completed in approximately 12 to 15 minutes and consists of 25 questions grouped into 5 domains: emotional distress, sleep disturbance, food/drink problems, vitality, and physical/social functioning [16]. The QOLRAD also has been translated and cross-culturally validated into languages including French, German, Spanish, Italian, and French Canadian [24]. The QOLRAD was administered at the randomization (baseline) and week-8 (final) visits. Responses were based on a 1-week recall period and scored using a 7-point Likert response scale (1 = all of the time/a great deal; 7 = none of the time/none at all); higher scores indicated better QOL. Administration of the QOLRAD was standardized, and procedures were used throughout the study to enhance patient compliance in correctly completing the QOLRAD, such as quiet, privacy, and administration before other examinations.

### Statistical analyses

Data from patients in both treatment groups were pooled for analysis, and between-group comparisons were not made. Mean total scores and individual domain scores were calculated at baseline and final visits and compared using the paired *t *test. Mean change in score between baseline and the final visit was considered clinically significant if 0.5 or more units [9]. This minimally clinically significant change was established based on findings from a previously conducted study assessing the reliability and responsiveness to change over time of QOLRAD [9]. Missing item responses were replaced by the mean value of completed items in that domain if 50% or more items in that domain had been completed.

A post hoc assessment was conducted to compare baseline QOLRAD scores from adolescents in this study with those of adults in the previous QOLRAD validation study [16]. Though the methods and primary results of the QOLRAD validation study were published previously, baseline scores had not been reported previously [16]. Briefly, the adult study was an international, multicenter psychometric evaluation in which 759 adult patients with chronic or recurrent upper gastrointestinal symptoms completed the QOLRAD. In the analysis presented here, mean baseline QOLRAD scores from each of the 5 domains were calculated according to baseline heartburn severity (mild, moderate, severe). Differences in QOLRAD scores 0.5 or more units between adult and adolescent patients were considered to be clinically significant.

## Results

Of the 149 patients randomized, more were girls (59.7%) and white (83.2%) and the mean body mass index was 23.5 ± 4.9 kg/m^2^. Of these 149 patients, 134 completed the QOLRAD at the baseline and final visits.

**Figure 1:**
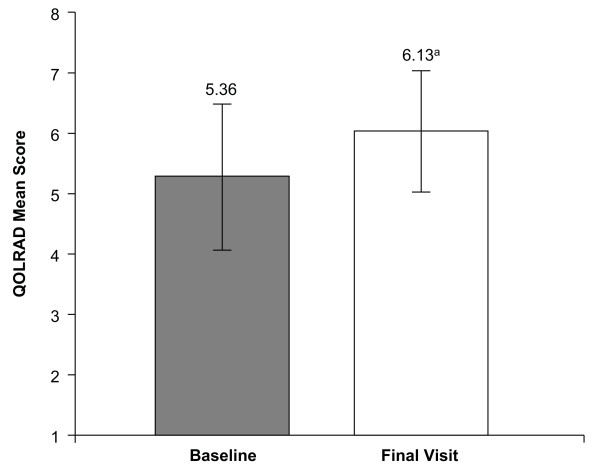
**Quality of Life in Reflux and Dyspepsia questionnaire (QOLRAD) mean total scores**. QOLRAD mean total scores ± standard deviation at baseline and final visit (both based on a 1-week recall) (n = 134). Higher scores indicate better quality of life. ^a^*P *< .001 vs baseline.

**Figure 2:**
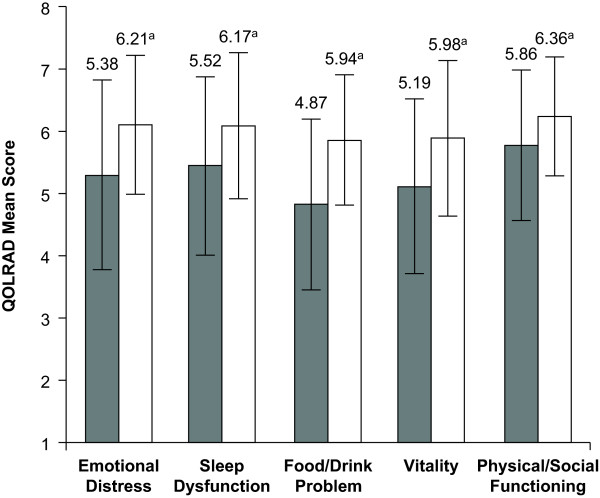
**Quality of Life in Reflux and Dyspepsia questionnaire (QOLRAD) mean domain scores**. QOLRAD mean domain scores ± standard deviation at baseline and final visit (both based on a 1-week recall) (n = 134). Higher scores indicate better quality of life. ^a^*P *< .001 vs baseline.

Baseline QOLRAD mean total scores (Figure 1) and mean domain scores (Figure 2) indicated that GERD had a negative effect on the HRQOL of these patients compared with normal adults. The QOLRAD domains most negatively affected by GERD were vitality, emotional distress, and problems with food/drink (Figure 2).

QOLRAD mean total scores and mean domain scores were improved significantly from baseline values after 8 weeks of esomeprazole therapy (*P *< .001) (Figures 1 and 2). Mean changes from baseline values in the QOLRAD total and domain scores met or exceeded the established adult QOLRAD minimum clinically significant difference standard of 0.5 units (Table 1) [9].

**Table 1:** Mean changes in QOLRAD total and domain scores from baseline to final visit (n = 134).

QOLRAD Domain	Change in Mean Scores^a^	Number of patients who significantly improved, n (%)^b^	Number of patients who significantly worsened, n (%)^b^
Emotional distress	0.83	67 (50.0)	10 (7.5)
Sleep disturbance	0.65	61 (45.5)	13 (9.7)
Food/drink problems	1.07	85 (63.4)	9 (6.7)
Vitality	0.79	73 (54.5)	17 (12.7)
Physical/social functioning	0.50	56 (41.8)	13 (9.7)
Total score	0.77	-	-

Abbreviation: QOLRAD, Quality of Life in Reflux and Dyspepsia questionnaire.

^a^*P *< .0001; ^b^Significant improvement and significant worsening defined as a change in score of 0.5 from baseline.

At baseline, QOLRAD scores were similar in adolescents and adults with mild heartburn (Table 2). However, the differences in baseline QOLRAD scores in the domains of emotional stress, sleep disturbance, and vitality between adolescents and adults with moderate heartburn were clinically significant (scores varied by ≥0.5 units). In addition, differences in baseline scores for the sleep disturbance and vitality domains between adolescents and adults with severe heartburn were clinically significant (Table 2).

**Table 2:** Comparison of baseline QOLRAD scores in adolescents and adults based on heartburn severity.

	Heartburn Severity at Baseline					
			
	Mild		Moderate		Severe	
	
QOLRAD Domain	Adolescent	Adult^a^	Adolescent	Adult^a^	Adolescent	Adult^a^
Emotional distress	5.4	5.2	4.9	4.1	3.5	3.5
Sleep disturbance	5.4	5.4	5.0	4.4	4.8	3.7
Food/drink problems	4.6	5.0	4.3	4.0	3.8	3.5
Vitality	5.1	5.0	4.5	3.9	4.0	3.2
Physical/social functioning	5.9	6.0	5.5	5.1	4.7	4.3

Abbreviation: QOLRAD, Quality of Life in Reflux and Dyspepsia questionnaire.

^a^Data from adults are derived from a previous study by Wiklund et al [16].

## Discussion

In this study, the burden of illness in adolescent patients with GERD was measured by a diseasespecific HRQOL instrument, QOLRAD, previously validated in adults [16] and used for the first time here in adolescents. Our results demonstrated that GERD has a negative effect on HRQOL in adolescents. These results are consistent with those of a recent study by Tolia et al [15], which shows that, in children aged 2 to 18 years, GERD symptoms negatively affect the QOL of children and their parents.

Because no placebo control group existed in the study, it could not be determined how much of the change from baseline in QOLRAD scores was attributable directly to esomeprazole therapy. However, this study is the first to show an improvement from baseline in HRQOL in all QOLRAD domains (emotional distress, sleep disturbance, food/drink problems, vitality, and physical/social functioning) in adolescents after treatment with a PPI in a clinical trial. In addition, the trial was a safety study, and patients were randomized to different dose groups for this purpose; it was not designed to be a comparative dosing study of esomeprazole. Between-group assessments were not made, and pooling of the data allowed for a better estimate in comparison with QOLRAD data in adults. Despite these study limitations, the findings are consistent with improvement considered clinically significant (≥0.5 units) [9] and establish a benchmark for future QOL studies in children of different ages with GERD.

In adult patients, 4 weeks of treatment with the PPI esomeprazole has been shown to significantly improve QOLRAD scores from baseline [9,11,13,14]. In the study reported by Talley et al [9], after 4 weeks of treatment with esomeprazole 20 mg, esomeprazole 40 mg, or omeprazole 20 mg daily, changes from baseline in QOLRAD domain scores ranged from 0.81 to 1.43. This unit change for adult QOL was higher slightly than the changes from baseline reported in this study in adolescents (Table 1) [9]. Similarly, El-Dika et al [14] reported changes in domain scores after 4 weeks of treatment with esomeprazole ranging from 1.3 to 2.0. However, in a study reported by Pace et al [11], 4 weeks of treatment with esomeprazole 40 mg yielded domain changes ranging from 0.37 to 0.66, slightly lower than those observed in our study (Table 1). Moreover, studies in adults have shown that improved QOL is maintained with esomeprazole treatment through 6 months [11,13].

QOLRAD scores from adults in other studies are difficult to compare with scores from this study of adolescents because the change from baseline in QOLRAD scores in sleep disturbance and food/drink problems has been shown to be associated with baseline symptom severity (none, mild, moderate, or severe); therefore, the largest changes in QOLRAD scores occur in patients with more severe symptoms at baseline [25]. An appropriate comparison of the effect of GERD on QOL between adults and adolescents would be within the baseline level of symptom severity. Therefore, we compared baseline QOLRAD scores according to baseline heartburn severity from adolescents in this study with those from adults in a separate study [16]. For patients with moderate or severe heartburn, clinically significantly greater scores were observed for adolescents compared with adults for the QOLRAD domains of emotional distress (moderate heartburn only), sleep disturbance, and vitality, indicating that moderate or severe heartburn has a more negative effect on HRQOL in adults than adolescents. However, the effect of mild heartburn on HRQOL in adolescents and adults is similar.

## Conclusion

Clinically significant improvements in HRQOL from baseline values were observed in this 8-week trial of esomeprazole treatment for GERD, which suggests that esomeprazole treatment may reduce the negative effect of GERD on HRQOL, particularly in the domains of vitality, emotional distress, and problems with food/drink in adolescents.

## Competing interests

TG, VT, RBC, and BDG receive grant/research support from AstraZeneca; TG, VT, and BDG are consultants to AstraZeneca; BDG and VT are consultants to Wyeth; BDG is a consultant and speaker for TAP pharmaceuticals and on the Advisory Board of Santarus, Inc.; VT receives research support from GlaxoSmithKline and Wyeth; MI, BT, and JAC are employees of AstraZeneca LP.

## Authors' contributions

TG was involved in the conception and design of the manuscript, revising the manuscript critically for intellectual content, and final approval of the version to be published. VT was involved in the conception and design of the manuscript, acquisition of data, analysis and interpretation of the data, drafting the manuscript, revising the manuscript critically for intellectual content, and final approval of the version to be published. RBC was involved in acquisition of data, revising the manuscript critically for important intellectual content, and final approval of the version to be submitted. BDG was involved in the conception and design of the manuscript, analysis and interpretation of the data, revising the manuscript critically for intellectual content, and final approval of the version to be published. BT was involved in the conception and design of the manuscript, analysis and interpretation of the data, drafting the manuscript, revising the manuscript critically for intellectual content, and final approval of the version to be published. MI was involved in the conception and design of the manuscript, acquisition of data, analysis and interpretation of the data, revising the manuscript critically for intellectual content, and final approval of the version to be published. JAC was involved in the conception and design of the manuscript, analysis and interpretation of the data, revising the manuscript critically for intellectual content, and final approval of the version to be published.

## Pre-publication history

The pre-publication history for this paper can be accessed here:

http://www.biomedcentral.com/1471-230X/9/84/prepub
